# Delayed facial nerve palsy after vestibular schwannoma resection: risk factors, extent and prognosis

**DOI:** 10.1007/s00405-024-08883-8

**Published:** 2024-08-10

**Authors:** Miriam Simon, Laurenz Althaus, Manuel Burggraf, Angelika Albrecht, Jörg Schipper, Julia Kristin

**Affiliations:** 1 Department of Otorhinolaryngology, University Hospital Düesseldorf Head and Neck Surgery, Moorenstrasse 5, 40225 Düsseldorf, Germany; 2 Department of Trauma surgery and Orthopedics, Essen University Hospital, Hufelandstraße 55, 45147 Essen, Germany

**Keywords:** Vestibular schwannoma, Delayed facial nerve palsy, Facial nerve outcomes, Cranial nerve failure, Skull base surgery

## Abstract

**Introduction:**

Facial nerve palsies may develop during the postoperative period of microsurgical removal of vestibular schwannomas (VSs), even after normal facial function for days or weeks after surgery. The aim of this study was to identify the pathomechanism and predictive factors of delayed palsy.

**Material and method:**

The clinical data of 193 patients who underwent vestibular schwannoma surgery between 2012 and 2021 were retrospectively analyzed. A total of 134 patients were included. The patients showed intact facial nerve function up to 24 h after surgery. All patients (*n* = 20) with palsy from postoperative day 4 were included and collectively referred to as delayed facial nerve palsy (DFNP). Various factors were checked using a binomial regression analysis.

**Results:**

The mean age of patients with DFNP was 57.8 years (55% female, 45% male). 70% had VS with KOOS ≥ 3, and 60% underwent surgery via a translabyrinthine approach Among the 16 patients with DFNP-related neurotropic pathogens, 25% were seropositive for herpes simplex virus. Most patients (*n* = 9/20) experienced onset of palsy between postoperative days 6 and 10. Of the four variables included in the significance test, three were significant: KOOS ≥ 3 (*p* < .04), ipsilateral vestibular organ failure (*p* < .05), and age group (*p* < .03). After therapy, 100% of patients recovered almost complete facial nerve function.

**Summary:**

The parameters mentioned above (KOOS classification and ipsilateral vestibular dysfunction) could be proven risk factors for the occurrence of DFNP.

## Introduction

Facial nerve palsies (FNPs) may develop during the postoperative period of microsurgical removal of vestibular schwannomas (VSs), even after normal facial function for days or weeks after surgery. There are many studies on postoperative direct palsy, but delayed palsy has rarely been described in terms of its occurrence and pathomechanism.

In the literature, the time of onset is often distinct. Sampath et al. distinguished between delayed-onset and late-onset palsies (those more than one week after surgery) [1]. Delayed-onset palsy is thought to be due to postoperative inflammation, vasospasm and edema, whereas late-onset palsy is believed to be caused by herpes simplex virus reactivation [2–4]. Tawfik et al. defined delayed facial nerve palsy (DFNP) more generally as a palsy occurring on the day after surgery or later [5–8]. However, the pathophysiological mechanisms of these DFNPs are poorly understood. Opinions vary widely, and a consistent line from onset on the first postoperative day to an arbitrary and unspecified later time frame remains elusive [9]. 

The severity of FNP varies from mild dysfunction (House Brackmann HB II/III) to almost complete/complete paralysis (HB IV/V/VI). Preservation of facial nerve function is important to the patient, as reduced facial nerve function is directly associated with reduced health-related quality of life [10–12]. It should be emphasized, that FNPs affect mental health even more than physical health [13]. Particularly because of the medical, psychological and functional sequelae of facial nerve weakness, a considerable amount of attention has been given to the factors that have a major impact on facial nerve outcomes [1]. These efforts are mainly focused on FNPs occurring immediately during or after surgery. The causes, reasons or risk factors for developing DFNP are still largely unknown.

Therefore, the aim of our study was to identify possible risk factors for the development of DFNP. Patient-, tumor-, and procedure-specific risks were examined.

## Materials and methods

The clinical data of 193 patients who underwent vestibular schwannoma surgery at the Department of Otorhinolaryngology, University Hospital Düsseldorf, Germany, between 2012 and 2021 were retrospectively analyzed. Included were *n* = 134 patients ≥ 18 years of age. The patients were operated on via a translabyrinthine or retrosigmoidal approach and showed intact facial nerve function up to 24 h after surgery. All patients (*n* = 20) with palsy from postoperative day 4 were included and collectively referred to as DFNP. All patients (*n* = 20) had DFNP (occurring between 4 and 14 days after surgery with an HB score of II-V). One patient with DFNP was excluded due to missing MRI images in the clinic’s digital documentation system and lack of postoperative follow-up at the time of data collection for evaluation. A total of 59 patients with postoperative direct FNP were excluded.

The following factors were examined: sex, age, vestibular failure, hearing loss, tinnitus status, KOOS classification, surgical approach, side of the schwannoma, subtotal tumor resection, neurotropic pathogens, cerebrospinal fluid leakage and revision surgery [14]. In addition, the time of onset, duration of recovery, diagnostic procedures performed, and possible therapies will be recorded and discussed.

Microsoft Excel version 16.70 and IBM SPSS Statistics version 29 software were used for statistical evaluation of the data sets. Patients were dichotomized into age groups with a cutoff value of 50 years. Binary logistic regression was subsequently performed to examine the extent to which the potential risk factors contributed to the development of DFNP. The regression model was statistically significant, χ²(4) = 14.339, *p* = .006, with a very good explanation of the variance of Nagelkerkes = 0.86, according to the recommendations of Backhaus et al. (2003).

## Results

Among the 193 patients, 59 had postoperative direct FNP, 20 had DFNP, and 114 had no FNP. 59 patients with postoperative direct FNPs were excluded. A total of 134 patients (without palsy and with DFNP) remained and were included in our study.

Descriptive statistics (Table 1):

**Table 1:** Characteristics of the patients with delayed facial nerve palsy

characteristics	*n* = 20 (in %)
age ≤ 50 / > 50 years	6 (30%) / 14(70%)
♀ / ♂	11 (55%) / 9(45%)
right side / left side	15 (75%) / 5(25%)
translabyrinthine- / retrosigmoid approach	12 (60%) / 8(40%)
ipsilateral vestibular organ failure / hypofunction	4 (20%) / 5(25%)
cerebrospinal fluid leak	4 (20%)

The mean age of the patients with DFNP was 57.8 years (SD ± 15.1), and the median was 57 years. Patients without palsy had a mean age of 60.3 years (SD ± 13.1) and a median age of 61.5 years. Regarding the sex distribution, DFNP occurred in 55% of females and 45% of males and in 50.8% of females and 49.1% of males without palsy. The tumor was more frequently located on the right side in patients with (*n* = 15/20) or without (*n* = 62/114) palsy. All sizes of VSs were represented according to the KOOS classification (Table 2). 70% of patients with DFNP had VS with KOOS ≥ 3. Among patients without palsy, only 26% had a KOOS ≥ 3. A total of 56.7% of the patients (*n* = 134) underwent surgery via a translabyrinthine approach: patients with DFNP (60%) and patients without facial nerve palsy (56.1%). 5% of the patients with DFNP underwent subtotal tumor resection, whereas 28.1% of those without palsy underwent subtotal tumor resection. In total, 16 patients with DFNP-related neurotropic pathogens were diagnosed. 25% (*n* = 4/16) were seropositive for herpes simplex virus, 12.5% (*n* = 2/16) were seropositive for toxoplasmosis, and one patient (6.25%) was seropositive for neuroborreliosis. During the postoperative course, 4 patients with DFNP developed cerebrospinal fluid (CSF) leakage. Of these, the leak resolved spontaneously in 2 patients (*n* = 2 patients, 1 patient underwent CFS drainage, and 1 patient required revision).

**Table 2:** KOOS classification and its distribution

KOOS classification	*n* = 20 (in %)
KOOS 1	4 (20%)
KOOS 2	2 (10%)
KOOS 3	10 (50%)
KOOS 4	4 (20%)

A variable time of onset was observed in patients with DFNP. The earliest appearance of DFNP was observed 4 days after resection, and the latest appearance was observed 14 days after resection. Most patients (*n* = 9/20) experienced onset of palsy between postoperative days 6 and 10. Among these patients, 20.0% developed palsy ≤ 7 days after the surgery, and 80.0% developed palsy > 7 days postoperatively. The HB scores were as follows: II: *n* = 5/20, III: *n* = 4/20, IV: *n* = 5/20, V: *n* = 6/20, and VI: *n* = 0/20. After inpatient treatment with corticosteroids and antivirals, 19/19 (100%) of patients completely or almost completely recovered (HB score I-II) from DFNP within 12 months after surgery (Table 3). One patient was excluded at this point due to incomplete data. In one patient, improvement in facial nerve function was observed only after a delay of 12 months, from HB V to HB II.

**Table 3:** House-Brackmann-score before and after therapy

HB-Score	before therapy *n* = 20	after therapy *n* = 19
I	0	15 (78.9%)
II	5 (25%)	4 (21.1%)
III	4 (20%)	0
IV	5 (25%)	1 (5.3%)
V	6 (30%)	0
VI	0	0

### Exploratory data analysis

We tested whether there was a significant association between each of the collected putative risk factors and the development of DFNP. Of the 4 variables included in the significance test, 3 were significant: KOOS ≥ 3 (*p* < .04), ipsilateral vestibular organ failure (*p* < .05), and age group (*p* < .03). Age > 50 years had a positive prognostic effect with an odds ratio of 0.1 (95% CI [0.01, 0.68]), and preoperative ipsilateral vestibular failure had a negative prognostic effect with an odds ratio of 7.338 (95% CI [1.16, 61.70]). A KOOS stage ≥ 3 was significantly more likely to indicate DFNP, with an odds ratio of 9.24 (95% CI [1.36, 96.77]).

There was no significant difference in the following factors: sex, hearing loss, tinnitus, surgical approach, side, cerebrospinal fluid leakage, revision surgery, subtotal tumor resection or neurotropic pathogens.

## Discussion

Microsurgical resection, along with radiation (e.g., Gamma Knife, CyberKnife) and close observation with MRI (wait and scan), is one of the three therapeutic options. These options should be discussed with patients after the initial diagnosis of vestibular schwannoma as part of a participatory decision-making process.

In close anatomical proximity to the facial nerve, FNP is one of the risk factors that patients should be informed of prior to treatment, as it may occur regardless of treatment option. If this occurs, it can affect an individual’s quality of life. The incidence of direct postoperative FNP also varies widely in the literature but is reported to be approximately 30% [15]. Our results showed that 14.9% of patients had DFNP. In the study by MacDonald et al. (2022), 10.8% of the 288 patients examined had DFNP [12]. Older studies by Lalwani et al. (1995) and Grant et al. (2002) reported that the incidence of DFNP ranged between 5.0% and 30.0%, and our 14.9% is in this range [16, 17]. According to Lerner et al., the number of patients who develop postinterventional FNP following gamma knife irradiation is approximately 5.3% (*n* = 7/133), depending on the radiation dose [18]. The incidence of FNP in patients who underwent a “wait and scan” procedure has not been clearly reported.

Regarding the classification according to the time of occurrence (Tawif et al.), there were no patients with DFNP after 2 or 3 days. The first patient developed DFNP after 4 days. However, Sampath et al. suggested a subdivision based on a probable pathomechanism: delayed palsy or late-onset palsy [19]. Postoperative complications such as edema and vasoconstriction and late onset with questionable viral reactivation were considered delayed facial palsy. Because palsy tended to develop 4 days after surgery and the sample size of the study was larger, we were able to define DFNP, and it is possible to include both potential pathomechanisms and to increase the size of the cohort of patients for a better evaluation.

The common classification of FNP severity is the HB score: I (normal function) to VI (complete paresis). We found that most patients initially had an HB score of II-V. 100% of patients completely or almost completely recovered (HB score I-II) from DFNP within 12 months, and 78.9% had an HB score of I and thus complete recovery without contractures or synkinesis. This favorable prognosis can also be confirmed by comparing the prognoses reported in current studies. Carlstrom et al. described a recovery rate of 100.0% (complete to almost complete) for HB I-II in 60 patients. However, rather rigid criteria, such as a time frame of occurrence between 5 and 30 days after surgery, were used to define DFNP [9]. On the other hand, Yawn et al. reported a recovery rate of 80.0% for HB I-II [20]. Notably, almost all the studies reported a recovery rate of HB between I and II, regardless of the possible presence of spasms or synkinesis at a score of II. A comparison of DFNP after microsurgery and idiopathic palsy showed that the outcomes may be similar. Among idiopathic patients (known as Bell’s palsy), 71% experienced complete recovery of facial muscle function (i.e., 61% of people with complete palsy and 94% of people with partial palsy). Spontaneous regeneration is possible within a month and affects most patients. However, approximately 30% of patients experience delayed or incomplete recovery, which has long-term effects on quality of life and self-esteem [21]. 

Despite the good recovery rate of patients with DFNP, it is still unclear which risk factors may lead to paresis. Therefore, an exploratory data analysis was performed to analyze tumor size and preoperative vestibular failure as risk factors. These are discussed below. An average age of more than 50 years has a positive prognostic influence on the development of DFNP. Patients are often diagnosed between the ages of 50 and 60 [22]. Vestibular schwannomas often grow slowly, so the first clinical symptoms may not appear until later in life. The European Academy of Otology and Neuro-Otology (EAONO) defines growth as a change in size of more than 3 mm on two consecutive MRI scans within one year [23]. This is quite different from, for example, highly malignant and destructive fast growing glioblastomas [24]. The higher incidence in patients older than 50 years is consistent with current literature. However, the present study cannot explain why older age should be a protective factor against the occurrence of DFNP.

The size of the vestibular schwannoma was graded according to the KOOS. Among patients with palsy, 70.0% had a vestibular schwannoma with a KOOS ≥ 3, and 26.3% of patients without palsy had a KOOS ≥ 3. According to our subgroup analysis, patients with a KOOS ≥ 3 had a significantly higher likelihood of developing DFNP. Increasing tumor size and associated additional aspects, such as increased surgical complexity, possibly irritation of the nerve and increased susceptibility to traumatic or traction injury, have already been discussed in the current literature. Increasing tumor size has previously been associated with worse facial function outcomes after surgical resection of vestibular schwannomas [15, 25–32]. In contrast, Carlstrom et al. reported no significant effect of tumor size on the severity of DFNP in their study group. He found that patients with DFNP had a higher rate of total tumor resection and retrosigmoid surgery, whereas other authors found no difference in these characteristics [9, 17, 33, 34]. MacDonald et al. reported a significantly larger maximum tumor diameter of approximately 5 mm in patients with DFNP. There were no other significant correlations with surgical approach or extent of resection [12]. This finding is consistent with our findings, as we did not find any significant differences in surgical approach or residual tumor status regarding the occurrence of DFNP.

Preoperative ipsilateral vestibular failure had a negative prognostic effect on the development of DFNP. This means that the probability of occurrence of DFNP after surgery is significantly higher.

Generally, dizziness is a symptom that is discussed preoperatively or after surgical resection. In a review by Saman et al., 50–69% of patients experienced dizziness preoperatively, and 64–90% experienced dizziness immediately after surgery. There were no significant differences in terms of approach or tumor size [35]. 

This negative prognostic influence could be explained in a similar way to that of the KOOS classification. A larger tumor or a displacing site with more extensive growth and more complex surgical resection is more likely to lead not only to vestibular dysfunction but also to failure with severe vertigo attacks until central compensation is achieved.

In our study, 25% (*n* = 4/16) of patients with DFNP were seropositive for herpes simplex virus, but this was not a significant risk factor. Treatment with aciclovir is recommended according to established guidelines, but the limitations of serology regarding seroconversion, lack of secretory vesicle smear and lack of CSF puncture must be considered [36]. 

## Summary

In conclusion, the parameters mentioned above (KOOS classification and ipsilateral vestibular dysfunction) could be proven risk factors for the occurrence of DFNP, especially a larger tumor size. In the future, a uniform definition of DFNP is needed, and potential risk factors should be included in patient information and treatment planning.

Patients should also be informed about good near 100% recovery after delayed palsy.
